# International primate neuroscience research regulation, public engagement and transparency opportunities

**DOI:** 10.1016/j.neuroimage.2020.117700

**Published:** 2021-04-01

**Authors:** Anna S. Mitchell, Renée Hartig, Michele A. Basso, Wendy Jarrett, Sabine Kastner, Colline Poirier

**Affiliations:** a Department of Experimental Psychology, University of Oxford, Oxford, United Kingdom; b Centre for Integrative Neurosciences, University of Tübingen, Tübingen, Germany; c Max Planck Institute for Biological Cybernetics, Tübingen, Germany; d Department of Psychiatry and Psychotherapy, Central Institute of Mental Health, Medical Faculty Mannheim, Heidelberg University, Mannheim, Germany; e Fuster Laboratory of Cognitive Neuroscience Department of Psychiatry and Biobehavioral Sciences UCLA Los Angeles 90095, CA United States; f Understanding Animal Research, London, United Kingdom; g Princeton Neuroscience Institute & Department of Psychology, Princeton University, Princeton, United States; h Biosciences Institute & Centre for Behaviour and Evolution, Faculty of Medical Sciences, Newcastle University, United Kingdom

**Keywords:** Primates, Housing standards, Neuroimaging, MRI, Welfare, Culture of care, Public engagement

## Abstract

Scientific excellence is a necessity for progress in biomedical research. As research becomes ever more international, establishing international collaborations will be key to advancing our scientific knowledge. Understanding the similarities in standards applied by different nations to animal research, and where the differences might lie, is crucial. Cultural differences and societal values will also contribute to these similarities and differences between countries and continents. Our overview is not comprehensive for all species, but rather focuses on non-human primate (NHP) research, involving New World marmosets and Old World macaques, conducted in countries where NHPs are involved in neuroimaging research. Here, an overview of the ethics and regulations is provided to help assess welfare standards amongst primate research institutions. A comparative examination of these standards was conducted to provide a basis for establishing a common set of standards for animal welfare. These criteria may serve to develop international guidelines, which can be managed by an International Animal Welfare and Use Committee (IAWUC). Internationally, scientists have a moral responsibility to ensure excellent care and welfare of their animals, which in turn, influences the quality of their research. When working with animal models, maintaining a high quality of care (“culture of care”) and welfare is essential. The transparent promotion of this level of care and welfare, along with the results of the research and its impact, may reduce public concerns associated with animal experiments in neuroscience research.

## Highlights

• Identifying the international standards for research ethics and regulations concerning non-human primates (NHPs).
• Introduction of an international animal welfare and use committee (IAWUC).
• Implementation of standards for animal welfare and care in research facilitates global collaboration efforts.
• International collaborations can improve the standards of animal welfare and care.
• Transparency in scientific research with NHPs influences public opinion and aids in public engagement.

## 1 Introduction

International collaborations are critical to rapidly advance scientific endeavours, as evidenced by successful international collaborations developed in response to coronavirus SARS-CoV-2 (Covid-19). For neuroscience, international collaborations are also vital in our quest to determine how the brain functions in normal and abnormal states. However, international collaboration can sometimes be hindered by national differences in welfare standards governing the use of non-human primates (NHPs). Despite the extensive ethical approval process and oversight surrounding NHP research within individual countries across the globe, there remains no common set of international regulations for NHP welfare in research that is comparable to the Declaration of Helsinki for human research. The lack of an international set of standards was highlighted as an impediment to fostering and enhancing international collaborations in the most recent PRIMatE Data Exchange (PRIME-DE) Consortium report (Milham et al., 2020).

Forging, and agreeing upon, acceptable common ethical and welfare standards used in NHP neuroscience research is necessary to support the researchers involved and to begin establishing vital international collaborations. The PRIME-DE Consortium community would like to address this challenge going forward. We believe that determining what are acceptable common standards, which do not compromise the welfare and care for the animals, nor scientific ethics, especially as applied to NHP neuroimaging research, will help establish international guidelines.

For our community, clear benefits are derived from data sharing, in particular an increase in statistical power (Button et al., 2013), for both existing NHP datasets and future ones. To protect the researchers involved in data sharing, additional transparency about the ethical and welfare standards applied to the NHPs involved in collecting these datasets is imperative. Transparency can be increased by including the identification of relevant regulatory bodies that provide the research approval, and references to published ethical and welfare standards followed in the experimental protocols. Further, providing details about current housing conditions and early life experiences of the NHPs involved in the studies is also invaluable (Poirier et al., 2021).

In essence, this article takes steps to identify a common ground in regulations, guidelines, oversight, and welfare standards applied to NHP neuroscience research, so we can move forward with developing international collaborations and catalyze change to benefit science and animal welfare. However, this article does not provide comparisons between countries or institutions regarding the time frames for approval, or level of details in protocols and procedures, that are required to fulfil the legal requirements for obtaining permission to conduct neuroscience research involving NHPs. We first clarify when and why NHP animal models are used in neuroscience research; then we identify the regulations for animal research across different countries and present the common international standards in place concerning NHP research ethics and regulations. In our article we do not suggest that these common standards are sufficient for international collaborations to proceed. Rather, this needs to be established by others (see below and Section 7). After setting out these common, but not minimum or minimal, standards in Section 4, we highlight many known examples of efforts to identify best practice and improve neuroscience research involving NHPs. For instance, neuroscientists are contributing empirical evidence that demonstrates improvements to standards of welfare and care for the NHPs involved in neuroscience research. Additionally, we explain some effective ways that neuroscientists, institutes, funders, and non-governmental organizations are engaging with the public and being more transparent about NHP animal models in research. Finally, in Section 7, to forge ahead in our endeavours to establish international collaborations in NHP neuroscience research, we propose the implementation of an International Animal Welfare and Use Committee (IAWUC) to provide a vital role in facilitating international communication and transparency on the range of animal care and welfare standards worldwide. With this, the IAWUC would provide advice to the relevant institutions’ regulatory bodies that authorize NHP research to ensure that institutions, funders, neuroscientists, and the NHPs are all safeguarded with these collaborations.

## 2 Non-human primate (NHP) animal models used in scientific research

Animal research, including NHP models, constitutes a vital part of our daily lives. This has been evidenced now, perhaps more so than ever, by the need for animal models, including NHPs, in the development of vaccines and antibody testing for Covid-19. With an animal model, scientists can model some process, mechanism, or aspect of human disease in an animal species, rather than trying to model the entire human experience.

NHP models (i.e., Old World macaques and New World marmoset monkeys) have played a key role in vaccine development and antibody testing (European Animal Research Association). The genetic, anatomical, physiological, and behavioral proximity of the monkey to the human makes them the best available animal model for certain topics, including neuroscience research (Phillips et al., 2014). Monkeys exhibit several skilled responses typical to primates, including humans (Friedman et al., 2017; Lemon, 2018; Nelissen et al., 2018). They also have similar binocular (Poggio and Fischer, 1977) and color (Merigan, 1989) vision to humans, and a comparable auditory system (Petkov et al., 2006). NHP models are, thus, particularly useful for studying sensory responses, grasping motor control, and prosthetic development, amongst others.

Moreover, research with NHP models has been effective in developing treatments for several human brain disorders, such as Parkinson's disease and dystonia, motor neuron disease, dementia, stroke, and neuropsychiatric disorders (Buffalo et al., 2019; Bernardi and Salzman, 2019; Capitanio and Emborg, 2008; Friedman et al., 2017; Lemon, 2018; Roberts and Clarke, 2019). As brain disorders are a major contributor to the burden of disease across the globe and a significant public health challenge (WHO, 2006), an understanding of such disorders and translating this understanding into new therapies and biomarkers are fundamental.

An example highlighting the use of NHPs as an animal model is with the study of abnormal behavioral phenotypes, resulting from genetic modifications related to human psychiatric disorders. Developmental and communication disorders, such as Autism Spectrum Disorder (ASD), have typically been studied in transgenic rodent lines manufactured to help researchers characterize behavioral phenotypes at the cellular and molecular levels (Schmeisser et al., 2012; Won et al., 2012). However, recalling the behavioral proximity of NHPs to humans, social interactions and group behavior may be studied in NHPs with greater relevance to humans. Recent advancements have led to a successful NHP model for ASD (Tu et al., 2019; Zhou et al., 2019). The use of the NHP model in this context can bridge the gap between rodents and humans (Bauman and Schumann, 2018), helping to translate the neurobiological underpinnings of behavioral phenotypes to humans.

Major differences in primate vs. non-primate brain organization and connectivity (Ding, 2013; Joel and Weiner, 2000; Ventura-Antunes et al., 2013) mean that more advanced, cognitive functions are better to be studied in primate models. NHPs can be trained to perform complex cognitive and behavioral tasks designed to study cognition and higher-order brain functions (e.g., learning, memory and recognition, visual attention, and decision-making). While brain function related to cognition and behavior are studied in humans and other species, NHP models allow for invaluable in vivo physiological recordings and/or the use of discrete brain perturbations during complex cognitive tasks, affording neuroscientists the opportunity to probe fundamental neural responses and determine the impact on an animal's cognition and behavior (Chakraborty et al., 2016; Mitchell et al., 2007; Pelekanos et al., 2020). Parallel studies applying the same task (i.e., attention) and methods (i.e., intracranial electrophysiology) in both humans (Helfrich et al., 2018; Martin et al., 2019) and NHPs (Fiebelkorn et al., 2018; Saalmann et al., 2012) are invaluable for deriving a comparative assessment of higher-order brain function. Primates are also crucial for the validation of non-invasive research methods used in human neuroscience. For example, neuroanatomy in monkeys, a gold standard approach (Carmichael and Price, 1995; Craig et al., 2014; Yeterian and Pandya, 1985), helps support neuroimaging studies on human brain connectivity (Van Essen et al., 2019). To curate comparative datasets, the Brain Initiative has worked to combine both human and monkey brain research in a publicly available manner.

**Fig. 1:**
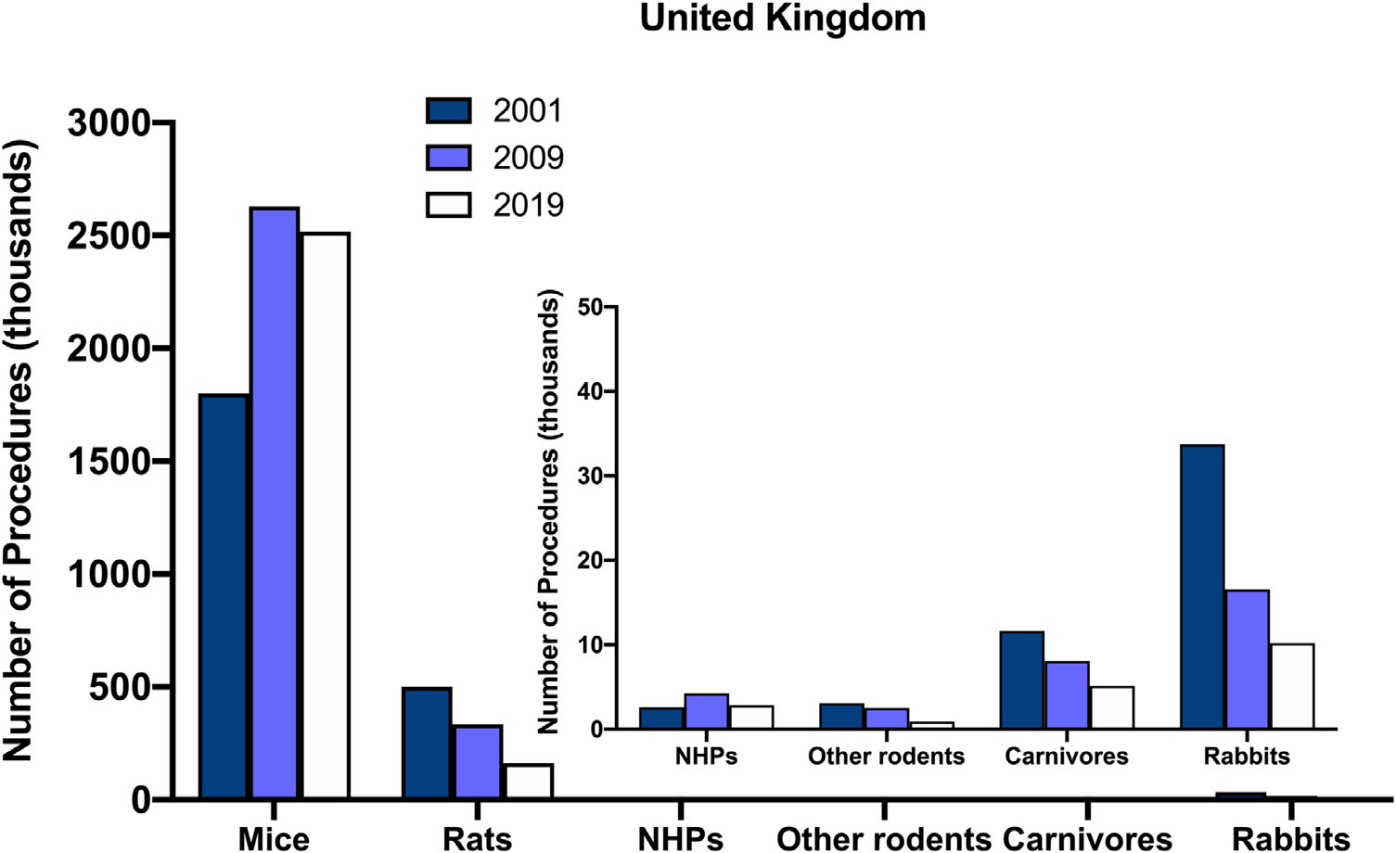
Regulated scientific procedures by species of animal (not all species included) conducted in the United Kingdom in 2001, 2009, and 2019. Insert: Smaller y-axis scale for NHPs, other rodents, carnivores, and rabbits as the overall numbers of procedures in thousands are comparatively small when presented alongside mice and rats. These data are made publicly available annually by the UK Home Office.

The use of NHP models in biomedical research, which includes the number of NHPs used in virology and immunology studies as well as those used in neuroscience, is comparatively small (see Fig. 1), yet it remains vital. For instance, statistics from the past two years showed that NHPs constituted just 0.11% of all animals used for scientific purposes in Germany (German Federal Ministry oFederal Ministry of Food andAgriculture,2019), and 0.08% for that in the UK (Home Office, 2020). Independent international reports commissioned by governments and funding bodies continue to indicate that animal research, including NHP models, cannot be abolished at this time without hindering scientific and medical progress (Bateson et al., 2011; EU Parliament, 2015; Friedman et al., 2017; SCHEER, 2017; Weatherall, 2006). It may even be the case that more monkeys need to be used in the near future, assuming appropriate standards of welfare, regulatory, and ethical considerations are met (Mitchell et al., 2018). Additional NHP models may be needed to provide fundamental understanding about the human brain that is not achievable using rodents or other mammalian species. NHP models may also aid in the investigation of diseases using new technologies or techniques (Singh et al., 2021). For example, NHP models may be used to provide insights using CRISPR-Cas9 techniques (Doudna and Charpentier, 2014), while specific transgenic monkey models may continue to be engineered to investigate human genetic disorders (e.g. Liu et al., 2014; Park et al., 2016; Zhou et al., 2019), and novel methodologies combined in NHP models may help progress study of neurodegenerative diseases (e.g. Beckman et al., 2019). While particular animal models are used in our endeavors to identify different aspects of human diseases, a rigorous and robust review of the most appropriate animal model is always determined via regulatory committees. Combining research that incorporates the most appropriate species allows for a multi-level and -layer approach to collecting the evidence to address specific scientific questions.

In summary, NHP models, along with other species are used to investigate specific scientific questions. Many advances in our understanding of brain function have resulted from the use of animal models and through the sharing of research outcomes, involving many species of animals, including NHPs. For example, NHP animal models helped develop successful treatments for amblyopia, which affects 4% of children around the world (Kiorpes, 2019) as well as effective treatments for Parkinson's disease, which affects about 1-in-500 people over the age of 50 years living in the UK (for review see Goldberg, 2019). Appropriate harm/benefit analyses should be applied to proposed future international collaborations involving NHP research models, to determine the extent of the harms and how harm can be mitigated with appropriate, humane interventions and end-points. As an added advantage to international collaborations involving animal models, the standards of welfare amongst all parties involved will improve by sharing best practices and the latest refinement successes (Prescott and Poirier, 2021). Preventing strictly regulated international collaborations in the future would only be a step backwards for both advancing standards of animal welfare and care, and our scientific endeavors.

## 3 Regulations for animal research across different countries

As stated in the most commonly referenced regulatory standards, “all who care for, use, or produce animals for research, testing or teaching must assume responsibility for their well-being (*Guide for the Care and Use of Laboratory Animals*, 2011).” The 8th edition of this guide, cited 10,895 times, is recognized internationally for setting standards for animal care and use. This *Guide* states that “both researchers and institutions have affirmative duties of humane care and use” of research animals, which is later defined as “those actions taken to ensure that laboratory animals are treated according to high ethical and scientific standards (NRC, 2011).” This *Guide* further states that “it is the institution's responsibility to put into place policies, procedures, standards, organizational structure, staffing, facilities, and practices to ensure the humane care and use of laboratory animals throughout the institution (NRC, 2011).”

This Guide (NRC, 2011) may serve as an international benchmark for countries with well-developed animal-based research programs. However, it must be noted that there are varying approaches in different countries to the use of animals for research, testing and teaching purposes. Why is that the case though? Each country has its own set of guidelines or regulations that is commensurate with national customs and local practices. Such differences in each country's guidelines can constrain collaborative efforts and/or contributions to global initiatives. At times, there is more involved than just ethics. For instance, there is the issue of regulatory burden, where politics and bureaucracy often supersede ethics. One example is the United States Animal Welfare Act, which excludes rats, mice, and birds (USDA), in a likely attempt to limit additional financial and regulatory burdens on biomedical research and the required enforcement by an additional government body (NABR, 2002). Overall, regulatory approaches and welfare standards need to be evidence-based in all countries.

At the regional level, the European Union (EU) Directive 2010/63/EU states that animals have intrinsic value that must be respected and that animal welfare considerations should be given the highest priority and each use be carefully evaluated (European Commission 2010). Further, a recent Commission Implementing Decision (European Commission 2020a, European Commission 2020b) based on the Directive re-emphasized the principles of replacement, reduction, and refinement (the 3Rs) when using animals in research. To systematically and centrally document and evaluate 3Rs implementation, EU member states will be required to submit non-technical project summaries and retrospective assessments of authorized projects (Office of the European Union, 2020).

At the global level, the World Organization for Animal Health (OIE) comprises more than 180 member countries. Its mandate is to improve animal health and welfare worldwide using the internationally recognized eight guiding principles on animal welfare outlined in Chapter 7 of its Terrestrial Animal Health Code. These principles include the 'five freedoms' and incorporate the use of the 'three Rs' for animals involved in science. A gross overview of the current laboratory animal science policies and administration in China, Japan, and Korea (Kong and Qin, 2010; MacArthur Clark and Sun, 2020; Ogden et al., 2016) reveals regulatory bodies analogous to countries like the UK, US, and EU countries.

Regular reviews of each countries’ regulations and the infrastructure and systems that support them also occur (e.g. Institute of Medicine (US); National Research Council (US); International Animal Research Regulations: Impact on Neuroscience Research, 2012). The revised *International Guiding Principles for Biomedical Research Involving Animals* is the result of a partnership between the Council for International Organizations for Medical Science (CIOMMS) and the International Council for Laboratory Animal Science (ICLAS), which formed to update the *Guiding Principles* (2012, available on the OLAW website) Statement of Principles for the Use of Animals from over 330 professional societies, organizations, and countries.

Typically, the basis for determining the morality of work in animals is founded in utilitarianism – the morality of an action is determined by its consequences (e.g., where causing harm to animals is acceptable if it increases the well-being to a greater number of others, including other animals and humans). Another moral viewpoint applied to research with humans is deontology, from the philosopher Immanuel Kant, which purports that the morality of an action is determined by moral rules or laws that consider the individual's dignity and worth. Animal welfare researchers have recently proposed a 'deontology' approach be used to determine the morality of research involving NHPs (Carvalho et al., 2018). A harm/benefit analysis using the utilitarian approach weighs the consequences of using an animal model (harm to the animal combined with mitigating effects, compared to the benefits to the greater good of knowing this information), while also considering the most appropriate species and the statistically necessary number of animals involved. A utilitarian approach for considering neuroscience research involving animal models may consider the impact and burden of medical conditions that affect the brain, such as mental health, neurological disorders, substance abuse disorders, and self-harm. This impact and burden was recently assessed as equating to 19% of total disability adjusted health years across countries that represent the Americas (Vigo et al., 2019).

## 4 Common international standards for animal research ethics and regulations with NHPs

To be able to forge international collaborations, we must identify the common, highest attainable, standard that is currently applied to the ethical and welfare regulations of NHPs across the world. At the international level, this common, highest attainable standard is imperative, but not sufficient to ensure the safeguarding of all parties, including the institutions, funders, scientists, and animals involved. Thus, an oversight committee with a mandate to establish the common and requisite standards of welfare and care for international collaborations is also required, as we propose in Section 7. The authors hope that this paper will raise more awareness with the OIE about the current lack of internationally approved ethical and welfare standards for NHPs. We also hope that the OIE may provide a possible leadership role in facilitating the creation of an internationally recognized oversight committee for international collaborations.

The previously published Culture of Care document (Brown et al., 2018) details this mandate: “Many of the laws and guidelines surrounding animal care and use allow for the use of professional judgment (Klein and Bayne, 2007). This should not be interpreted to support a minimalistic approach that just meets the letter of the law, but instead should be applied to working with animals in a manner that strives to provide the best possible care for the animals, thus, producing the highest-quality scientific results (Medina, 2008). A culture of care often starts with an institutional mission and value statement that clearly states the institution's commitment to the humane care and use of animals (Phanuel Kofi Darbi, 2012).” The culture of care for the animals has vast implications on the training of personnel and handling of research animals, and on our ability to work together on the global stage.

For the regulation of animal research including NHPs, it is universally agreed that two components to oversight and governance are essential:

1) A locally based ethical oversight committee operating within the institution or university that provides a key role in reviewing and approving protocols and experimental procedures involving NHPs. In many countries, including the USA and China, this committee is referred to as an IACUC (Institutional Animal Care and Use Committee). In the UK and EU, a similar type of institution-based committee also provides the oversight and initial approval at the local level. However, these institution-based committees do have different approval processes and responsibilities, depending on the additional levels of local, state, and national regulation of animal research. Nevertheless, the committee members must include people with appropriate expertise, including primate veterinarians, animal care staff, scientists conducting animal/NHP research, and the lay public.

2) Animal facility inspections to ensure ethics, welfare regulations, and scientific work is carried out as detailed.

During the process of writing this manuscript, data were collected based upon two evaluative tables (see Supplementary Information). These data and other publications available online (e.g. German law and UK Home Office) helped inform us of the ethical and welfare regulations in countries that conduct animal research involving NHP models. This information provides an overview of the care and use of NHPs (macaques or marmosets) in some of the countries that permit NHPs to be used in neuroscience research, comprising Supplementary Table 1: Ethical regulations for conducting neuroscience procedures with NHPs, and Supplementary Table 2: Animal welfare, including information regarding housing regulations (see Supplementary Information).

From Supplementary Table 1, across the institutions that conduct neuroscience research work with animals, including NHPs, both of the key components (outlined above) to oversight and governance are implemented. For example, in the US and China, the local IACUC, based at the institute or university, reviews the scientists’ application for work with NHPs. In the UK, the Netherlands, France, and Germany, similar local animal welfare, use, and ethical review committees, based locally (e.g., township, institute or university), review and provide the initial approval of applications. In addition, further review and approval of applications is provided at the level of national committees in the UK and EU, following the 2010/63/EU Directive. In all countries, members of the committees consist of a good balance of people with specialist knowledge, including veterinarians, animal care staff, and scientists involved in NHP and animal research, as well as lay people. In addition, regular (typically annual) inspections are conducted by these IACUCs and institution-based local committees, and in the case of the UK and EU, by regional inspectors and national committees as well.

From Supplementary Table 2, many common methods were reported regarding the care and welfare of NHPs involved in neuroscience research. For example, all NHPs are given daily opportunities for enrichment, in the form of objects to manipulate, a variety of foods, and/or behavioral training or time in exercise rooms (play cages). In some labs, NHPs also have access to music, television to watch, or touchscreen computers attached to their home cages. In addition, all NHPs are housed with visual and auditory contact with conspecifics. Marmosets are housed in family groups.

Across the UK and EU countries, similar time frames are used to wean marmosets (6–8 months) and macaques (from 8 months) from their mothers. Breeding transgenic monkeys can involve shorter time frames for weaning. Researchers in the US and China have highlighted a need to raise some transgenic infants in nurseries from birth onwards (Chan and Yang, 2009; Chan et al., 2014; Chen et al., 2012). In Japan, transgenic marmosets may be weaned at 3 months of age (Tomioka et al., 2017).

Regular weighing of the animals functions as a common, readily practiced measure of the monkey's health and wellbeing. While on protocols, monkeys may be weighed up to daily with no longer than 14 days between sets of weights. Clear guidelines and definitions of the amount of weight loss are provided - with veterinary examinations carried out if an animal's weight drops between 10-15% from its original weight, while an animal is removed from study if weight drops by 20% from original weight in China, similarly in the UK, EU, and USA with some minor differences.

Research protocols involving fluid and/or food control are similarly regulated across international NHP research labs, although within each research laboratory, the specific controls differ, in order to collect the scientific data from each particular animal (Poirier et al., 2021). There are guidelines provided for the minimum daily amount of fluid intake (20 mls/kg per day), and in some facilities there is a minimum amount of time that the NHP has free access to water (at least 3–6 h per 24 h and at least one day of free access per week). These guidelines have been outlined in Prescott et al. (2010).

One of the differences observed from Supplementary Table 2, although this is changing, is how NHPs are acquired for neuroscience research. In some countries where NHPs are used in neuroscience research, they must be acquired from purpose-bred facilities, where the animal health status and welfare are recorded. However, in China, prior to Covid-19, it was possible to use some NHPs that had been caught from the wild.

Another difference reported from Supplementary Table 2, is the sizing of the home enclosures and caging for the monkeys and marmosets. In the US, the sizing is dependent on weight of the monkey. For a monkey up to 15 kg, the floor size must be at least 6 square feet (0.56 m^2^) and the height at least 32 inches (0.81 m). A recent publication provides photographic images of NHP caging (McAndrew and Helms Tillery, 2016). In China, for an adult monkey, the cage sizing must be at least 1.0 m in height with 0.9 m × 0.7 m (0.63 m^2^) floor size. In the US and in China, monkeys also get access to playpens that are rotated amongst animals. Evidence-based research shows the use of playpens provide benefits to the NHPs (Griffis et al., 2013). In the UK, France, and Germany, cage sizing for rhesus monkeys aged 3 years and above must be at least 1.8 m in height, while the floor size must be at least 2 m^2^. Previous reviews have highlighted that the size of the caging used for housing research NHPs, wherever possible, needs to be large enough to allow for normal locomotion and displays of a normal behavioral repertoire depending on the species (e.g. Buchanan-Smith et al., 2004). However, this review also concluded that suitable cage furnishings with adequate complexity (e.g. perches or ledges located at different heights, and visual barriers to reduce aggression) are particularly beneficial (Buchanan-Smith et al., 2004). Importantly, these types of cage furnishings may be readily implemented in existing housing set-ups.

Another difference is that monkeys in the UK and EU are not allowed to live on a metal grid floor, it must be a solid floor (typically the floor of the room with special drains installed). This allows for the NHPs to forage on the floor for small grains and other foods, typically scattered amongst substrates, to provide additional enrichment opportunities.

Moving forward, it is advisable that scientists and others take actions to address differences in standards of care and welfare, while at the same time allowing NHP colleagues to embark upon international neuroscience collaborations, assuming that appropriate oversight and ethical regulations are in place. It is imperative that the ethical framework is not compromised in any way. To expand representation of different nations in neuroscientific research and to have oversight with an agreed set of standards (from those in place already in other countries), we suggest the implementation of an international advisory committee, an International Animal Welfare and Use Committee (IAWUC; see Section 7).

## 5 How scientists can contribute to improving standards of welfare and care for NHPs

There is a general agreement that standards of welfare and care regulation should be based on scientific evidence that documents the harms to the animals undergoing scientific procedures. However, currently, there is a paucity of scientific evidence on which policies are based and mandated. Fortunately, this is beginning to change with documenting the impact of protocols and procedures on NHPs and providing examples of best practice for NHP research. Further studies (see below) are still required though to continue identifying areas where the standards of welfare and care can be altered for the betterment of the NHPs and the science. Recently, DeGrazia and Beauchamp (2019) proposed a more comprehensive ethical framework, also considering the social benefits, to guide the *Principles for Animal Research Ethics* than the 'Three Rs' proposed by Russell and Burch (1959).

In many examples, detailed below, neuroscientists have conducted research as a side project to their main neuroscience research, in order to help capture and share best practices and refinements from their labs. This evidence also allows for more informed decisions to be reached about ethical regulations and the establishment of suitable proven techniques and protocols that support the welfare of laboratory NHPs involved in neuroscience research. Some of these studies also highlight methods that seemed promising, but not all have proven fruitful.

Recently, researchers have identified many different approaches and anatomical properties that have worked, and not worked, for optogenetics (Tremblay et al., 2020) and chemogenetics (Galvan et al., 2018) when applying rodent techniques to NHPs. In addition, these, and many other researchers have been working on methodologies that do work in NHPs (Magnus et al., 2019).

To evaluate the health and wellbeing of NHPs involved in neuroscience research quantitatively, several studies have assessed physiological (e.g. changes in cortisol) and behavioral measures in rhesus macaques involved in fluid control protocols and undergoing daily neuroscience procedures (Hage et al., 2014; Gray et al., 2016; Pfefferle et al., 2018). Others have assessed the relevance of using some behaviors as indicators of wellbeing (e.g. pacing behavior; Poirier and Bateson, 2017; Poirier et al., 2019). Recent refinements in the use of NHP cranial implants have been documented (Chen et al., 2017; Ortiz-Rios et al., 2018) and less invasive cranial implant procedures were found effective for neurophysiological recordings (Pigarev et al., 2009). As a result of a multi-institutional collaboration, a primate protective head cap has been developed and proven to be an effective 3Rs refinement that is used to protect the monkeys’ wound margins after cranial implant surgeries. Wearing the protective head cap dramatically reduced picking of wounds, eliminating the need to re-suture any wound margins (Perry et al., 2020). Basso and colleagues review successful methods for MRI scanning, including using effectively designed cranial implants (Basso et al., 2021). The recent development of the PRIME-RE platform (prime-re.github.io) provides a potential way to share NHP refinement approaches and protocols with international colleagues.

In addition, automated home cage training set-ups have been implemented in breeding centers that may help to pre-determine which NHPs are suitable for behavioral neuroscience research (Tulip et al., 2017). Another study has identified that using positive reinforcement training (PRT) alone is not effective for training all monkeys involved in neuroscience research, rather a combination of mainly PRT incorporating some negative reinforcement techniques proved more effective (Mason et al., 2019). Finally, other studies have investigated effective methods for non-invasively immobilizing the head of the NHP during some experiments involving awake MRI scanning (Hadj-Bouziane et al., 2014; Slater et al., 2016).

Clearly, further scientific studies are required to provide additional evidence and resources for others in the care and use of NHPs in neuroscience research. Future studies could include: welfare assessments of NHPs living in different housing conditions around the world, effective strategies for stress mitigation, and the most naturalistic working environments. National and international collaborations amongst NHP scientists can allow for the formation of larger sample sizes and promote sharing of best practice ideas. However, while many further, informative studies are possible, funding of this type of research is necessary and critical.

Currently, the burden of improving NHP welfare is mainly supported by researchers, who do not necessarily have the financial means to do something about it. Additional support is required from funders, institutions, and governments to fund improvements to staff training, infrastructure, and housing that impact animal welfare and continue to produce quality science. As many funders and governments have commissioned reports concluding that neuroscience research involving NHP models continues to provide scientific and medical benefits, this should provide a strong case for continuing with staff training and education, and for funding enrichment and refinement studies that provide empirical evidence about the implementation of suitable standards of welfare and care for NHPs involved in neuroscience research.

## 6 Effective means to engage the public and be transparent about NHP animal models in research

The use of animals in biomedical research is problematic for many people. We know from opinion polls that a large proportion of the public accepts animal research as long as it conforms to the prevailing regulations. For example in 2018, 68% of those polled in Great Britain agreed with the statement: “I accept the use of animals in scientific research as long as there is no unnecessary suffering to the animals and no alternative” (IpsosMORI). However, once we start scratching the surface of this acceptance, we find areas of opinion that suggest some conflict in people's answers. In this 2018 survey, a much smaller proportion (15%) of the British public found it acceptable that NHPs are used in research. Interestingly, when asked the same question about the use of animals in scientific research during the Covid-19 lockdown period in the spring of 2020, the percentage agreeing was 75% (Understanding Animal Research, 2020). Further, this second survey found that 73% of respondents answered ‘yes’ to the question “If scientists can only develop tests, treatments and potential vaccines for the Covid-19 virus by studying and testing on animals such as mice, dogs and monkeys, do you think that is acceptable?”

Nevertheless, the broad public support in the UK may well be reflected by attempts at transparency across different institutions. A growing list of institutes from different countries now have information detailing the use of animals as research models in studies that are conducted within their establishment or with their funding (e.g., the Medical Research Council, National Institutes of Health, Newcastle University, Oxford University, University of Mainz, and the Wellcome Trust). Providing such information in a transparent manner adds facts to the rational and emotional factors at play here. Most people rationally accept that animal research is necessary, but when asked to think about a particular animal being used in research, a more emotional response kicks in for a proportion of people who then say they cannot accept that certain species of animals are used. When it comes to primate research, we are all aware of the ethical dilemma of using such highly-intelligent animals: they are used because they are humans’ closest relatives and, in some cases, the only possible model for certain aspects of human physiology and cognition. But, we apply much stricter regulations and ethical considerations to the use of primates in research precisely because they are so intelligent and human-like. Rationally, we realize that no other animal model will work, but emotionally we empathize more with animals that are more like us.

Communication of any issue comprises rational and emotional elements. People need facts, but they also need information that addresses their worries and fears. And, it is far better to start that communication before anyone criticizes a particular research project or institution. If the public has some basic knowledge and understanding of a research program and its aims, they will be less likely to believe misinformation about it from a campaign group. If they know that the law requires certain standards, they will be less likely to believe stories purporting to show animal cruelty.

So, what can be done to help the public understand the reality of animal research and, in particular, the use of our closest relatives, NHPs? How can we describe the regulations behind the research studies and how such research, whether basic or applied, contributes to scientific understanding and innovation? The answer to those questions is engagement.

For many years, the only images and information in the public domain about animal research came from animal rights groups. They showed animals housed in horrible conditions, sometimes with seemingly distressing injuries. Usually these images had no provenance – no information about where and when they were taken. We have an uphill battle to counter people's mental image of “animal research”. To succeed we need to provide our own images, videos (podcast with Wendy Jarrett), and information about the reality.

There are several organizations around the world that help to explain why and how animals are used in research (e.g., Americansfor Medical Progress(AMP), European Animal Research Association, Foundation for Biomedical Research, Pro-Test Germany, Speaking ofResearch, and Understanding Animal Research). These supportive organizations advocate for animal research in different ways. Some organisations have individuals that do not conduct scientific research, some are a mix of those individuals and scientists themselves, and some are mostly scientists. One organization which consists of a committee of scientists who voluntarily advocate for science communication and transparency on animal research in their spare time is *Speaking of Research.* The grass-roots organization, *Pro-Test Germany*, for example, is mostly composed of early career researchers, whereas *Understanding Animal Research* is a mix of ex-research scientists and career-level public communicators. Patient advocacy groups also contribute to public outreach on animal research when appropriate. These groups pursue a wide range of activities from public outreach events to media interviews, online and print material, as well as training and consulting. In doing so, they often seek to represent or coordinate research institutions and the "scientific community" at large.

An unusual example drawn from these organizations is Pro-Test Germany, a decentralized non-profit run by about 80 volunteers with a focus on personal interactions. They offer visibility to their diverse peers (animal caretakers, research group leaders, graduate students, veterinarians), and work to enable them to share individual experiences, opinions, knowledge and doubts with their local community, either in person or anonymously. This includes street gigs, blogs, social media feeds, skill workshops, and advice to both employees and employers. Similar, but independent organizations have taken up the "Pro-Test" idea in other countries.

**Fig. 2:**
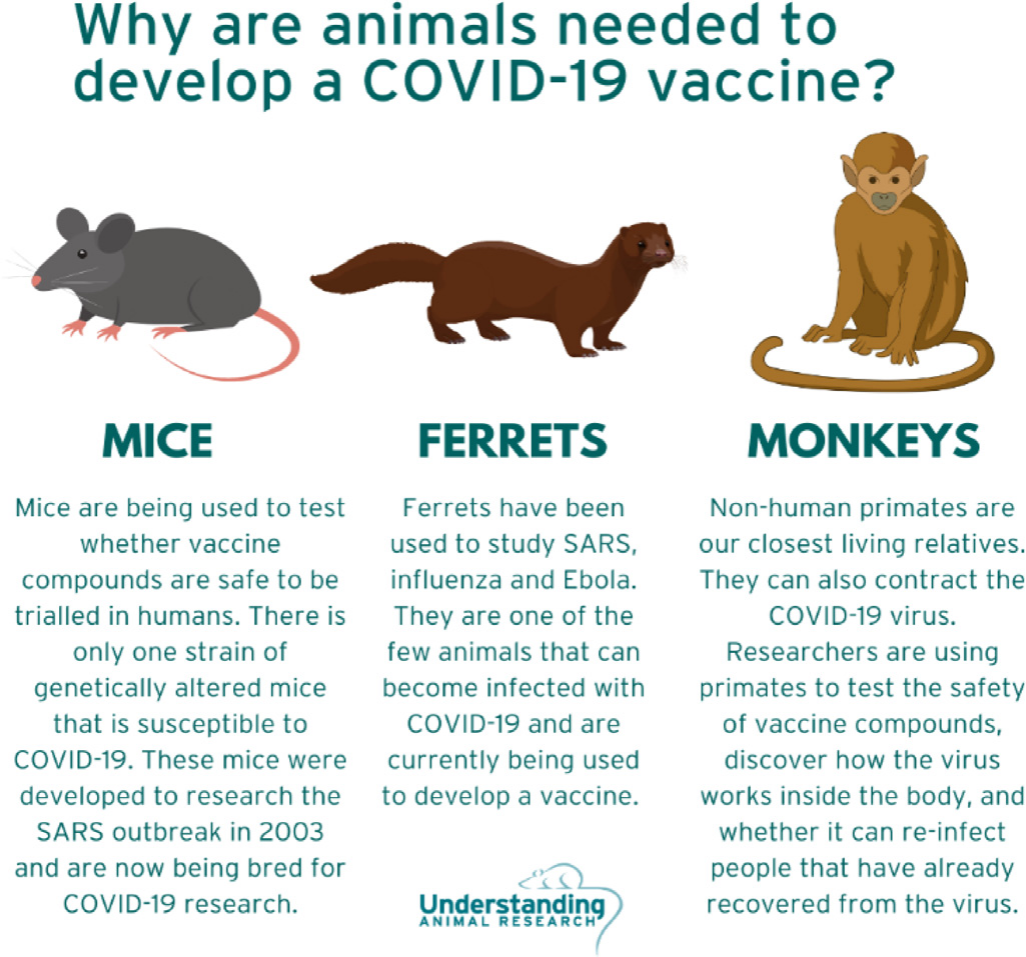
Infographic detailing why and how different mammals have been useful for research about Covid-19 from the Understanding Animal Research website.

It is important that animal researchers try to engage with the public if they can, even despite the vulnerabilities that come with public exposure. Research conducted at academic institutions are supported, at least in part, by public funding, but often there is a lack of communication between the lay public and the scientists. In today's digital age, one way for scientists to reach the public is through social media. Animal research advocacy groups, scientists, and individuals around the world can engage with thousands of people each day through posts on Facebook, Twitter, Instagram, and LinkedIn. Here, infographics explaining why animals are used in research have proven very popular (Fig. 2). Also gaining in popularity are podcasts, a few of which have dedicated episodes to animal research and public communication (e.g., Undark, the AMP-sponsored Lab Rat Chat). We also highlight some examples of outreach and media correspondence that scientists have recently done (e.g., on Facebook). However, for neuroscientists that conduct public engagement activities, they typically do so as a side project to their main neuroscience research, to help provide transparency about their research with the public. These additional efforts should be recognised in the extremely competitive scientific environment.

Furthermore, some countries’ biomedical research communities have taken steps to improve communication with the public by creating openness agreements. The first openness agreement, known as the *Concordat on Openness on Animal Research in the UK*, was published in 2014. By signing the Concordat, organisations that carry out or fund research using animals have committed to providing enhanced communications for the public and to create opportunities for people to find out more about the reality of animal research.

One project to come out of the Concordat was a 360-degree online tour of four UK research facilities (www.labanimaltour.org). This project features the University of Oxford's primate facility and allows anyone interested in finding out more about the use of NHPs in neuroscience to navigate themselves around the lab, seeing how macaques are housed and cared for, and how they go into a primate chair to prepare for their work, typically in front of a touchscreen. Here, it is clear that some animals have implants and head-posts. Information boxes and videos provide extra detail and explanation on what is going on, including interviews with researchers and animal care staff. The project was recently featured in *The Scientist*, highlighting the significance of such transparency efforts and their long-lasting impact. The idea for the UK Lab Animal Tour came from an online virtual tour of a French primate facility http://visite-animalerie.cnrs.fr/#/accueil/. The Primate Research Centers in the US and the German Primate Center also have public outreach websites offering a variety of resources (NPRC, DPZ).

One aspect of animal research that worries members of the public is its perceived secrecy – if no one talks about the research they are doing, could researchers potentially be hiding some of their research practices and procedures, or perhaps be duplicating research and using animals for research that has already been carried out somewhere else? This latter, relatively nuanced concern, came out during focus groups convened during the development of the UK Concordat. People are reassured when they hear of scientists sharing information and data. During the Zika and Ebola outbreaks, news outlets covered the fact that researchers were putting their data into the public domain for others to use in real time. Again, during the Covid-19 pandemic, much was made of the way scientists around the world shared their findings due to the potential of expediting vaccine discovery and treatments (Speaking of Research, 2020). In a similar way, the PRIME-DE Consortium (Milham et al., 2020) is encouraging researchers involved in NHP neuroimaging to unite efforts and share research data, ideas, and analysis methods with the hope that advances in our understanding about the brain and related diseases and disorders may be expedited. Unfortunately, for this community of neuroscience researchers, there is a barrier to fostering future international collaborations due to a lack of agreed upon regulatory oversight and standards of welfare and care of NHPs across the globe. To facilitate greater understanding about the use of NHPs in biomedical research, many organizations and scientists themselves are providing public-facing information with clear explanations (and in lay language) about what they are doing and why they are doing it. NHP neuroscientists are, therefore, confident that the aim of the PRIME-DE Consortium is one that the public will embrace and support.

## 7 Ways forward to international collaboration, proposing an international animal welfare and use committee (IAWUC)

As is apparent from our overview of responses in Supplementary Tables 1 and 2, there are some differences as well as many similarities in the international regulations and standards of welfare and care for the use of NHPs in neuroscience research. Some of these differences may well reflect differences in ethical values based on cultural differences. Consequently, when performing a harm/benefit analysis, the level of harm, combined with the mitigating interventions to NHPs, which is deemed acceptable for a due benefit will vary between countries. Cultural, and other differences between countries are apparent in the regulations applied to other species as well. Cultural differences should be allowed and accepted as long as they are suitably justified. However, at the same time, all of us need to strive to improve the standards of NHP care and welfare in order to achieve a common set of standards for the animals. For the former, empirical findings (see Section 5 for examples) can help guide regulatory approaches and welfare standards in an evidence-based manner.

Nevertheless, the same standards of regulation do not always apply to the same level in other species. For instance, hamsters, but not other rodents, are regulated in the US by the USDA. There are also differences between the US, China, and the UK, and EU regarding the regulation of rodents for neuroscience research. Even within what might seem to be similar cultures (e.g. Europe), where all member states are governed by the EU Directive 2010 for the care and use of animals (see Section 3), different countries can reach opposing conclusions when deciding what is the most ethically correct way forward for their country. For example, when the EU Directive was introduced, it implemented a ban on the use of wild-caught NHPs for research and instead imposed the use of purpose-bred NHPs in specially designed breeding centers. However, in Italy, despite having implemented the EU Directive, this country has banned purpose breeding of NHPs for ethical reasons. So, despite the current trend of increased harmonization, some cultural differences reflected in the national legislations are likely to persist. Therefore, it is not conceivably realistic to expect ethical and welfare standards to be fully harmonized.

Despite these differences, appropriately approved and regulated international collaborations must be allowed to forge ahead in NHP neuroscience research, without compromising the welfare and care of the NHPs or any ethical standards that may jeopardize the quality of the science. For NHP neuroscience researchers to be able to embark on international collaborations, we propose that an international committee be established. This committee would provide an oversight and advisory role, like an IACUC or Animals in Science (UK) committee, but its mandate would be to consider international neuroscience research collaborations. International committee members with relevant expertise would be proposed and elected. For example, there may be members from Society for Neuroscience (SfN), Federation of European Neuroscience Societies (FENS), the Japanese Neuroscience Society (JNS), the Chinese Society for Neuroscience (CNS), and individual European and British Neuroscience Societies as well as veterinarians with links to the European Primate Vet Association (EUPRIMVET), the US equivalent (AVMA), and Asian equivalents. Representatives with NHP animal welfare expertise will also be indispensable, such as members with NHP expertise from AAALAC and the NC3Rs.

The role of this independent committee would be to review applications and make suitably informed recommendations about proposed international collaborative ventures that do not risk the reputations of the funders, institutes, and universities involved, or the scientists. In doing so, this committee may help to formulate the minimal, yet highest attainable, standards required for successful multinational NHP collaborations. The proposed IAWUC is not meant to serve as another layer of bureaucracy, but rather as a facilitator of reputable international collaborations. Where NHP collaborations might currently be hindered, for example, between the UK and institutions in the US, or between the US or UK and institutions in China, or Japan, or in the EU, the IAWUC may provide impartial advice and support to mediate current obstructions. To move this proposal forward, the chair of the OIE, chairs of animal research regulatory bodies (e.g. the UK Home Office, national animal research regulators in the EU, and institutional IACUCs in the US, Japan, and China), funders of neuroscience research, and heads of neuroscience societies need to include this proposal on their forthcoming meeting agendas.

## 8 Conclusion

As has been evident from the international response to Covid-19, decision makers, advisors and leaders of countries monitored the effectiveness of different responses in order to implement health and safety procedures as well as treatment protocols within their own countries. Importantly, international collaborations amongst scientists have led efforts to rapidly sort potential vaccine candidates, provide antibody testing, and advances in our understanding. Synergistic efforts have accelerated the rate at which a vaccine could ever be produced. International concern for advancing our understanding and developing effective treatments and cures stands similarly for brain diseases, disorders, and neurodegeneration. Neuroscience is no exception. There are many institutions that support neuroscientific research, but are not able to support research using NHPs. Thus, investigators may want to ask specific experimental questions, yet are unable to do so because their facilities cannot support the animal model required. An NHP collaborative resource solves this problem by providing open access to data that can be mined to address new and exciting questions that might not otherwise have been asked.

As indicated in this article, we believe international collaborations involving NHP models are essential if our endeavors to understand the brain are to be successful. In the next 5 years, let us make it possible to work together and find commonalities that allow the differences in each country's standards of welfare and regulations to be workable and to consider the cultural differences on the value of NHPs. Establishing these international collaborative links will allow further sharing of data to optimize scientific excellence, reliability, reproducibility, and output. Most importantly, while sharing ideas and data, we must also share best practices that improve the standards of welfare and care of our animals.

While there are evident differences in standards of welfare and care based upon cultural values and diversity, researchers should acknowledge such differences and the potential effect it might have on scientific collaborations. If societal differences impede scientific progress and innovation, addressing the resulting issues are a step towards overcoming any problem. But, how can we overcome this predicament, so that collaborations can be implemented and maintained, and efforts towards scientific progress mutually benefit all parties involved? Overall, adopting a transparent approach that highlights and addresses these issues effectively is a basis for working towards improved ethical and welfare standards for the animals involved in neuroscience research.

## Declaration of Competing Interest

The authors declare no competing financial interests.

## Appendix Supplementary materials

Image, application 1
