# Supplementary material for: International primate neuroscience research regulation, public engagement and transparency opportunities
Source: Neuroimage. 2021 Apr 1;229:117700. doi: 10.1016/j.neuroimage.2020.117700 (PMC7994292; doi:10.1016/j.neuroimage.2020.117700)
Supplement: Supplementary file 1 [file mmc1.docx]

**Supplementary Information**

**Table 1A. Overview of ethical regulations for conducting neuroscience procedures in non-human primates (macaques and marmosets) in the UK.**

| **UK** | |
| --- | --- |
| What committees review and approve an NHP application? | |
| Local ethics committee | In the UK, all project licences authorising scientific research involving animals come under the Animal (Scientific) Procedures Act, 1986 and EU Directive 2010/EU/63. The Secretary of State at the UK Home Office provides authorization of the regulated procedures carried out on animals. The project licence is a legally binding document.  The local animal welfare and ethical review committee (AWERB) at each institution (e.g. universities and one NHP breeding centre) provides an ethical evaluation of the NHP project applications and a recommendation. If the AWERB does not approve the NHP project application, the application is not forwarded to the next stage.  Local ethical committees are at minimum composed of a Named Veterinarian Surgeon (NVS), Named Animal Welfare and Care Officer (NACWO), a scientist involved in animal research (NHP research when relevant), Establishment Licence Holder from the institution, a secretary, and public representatives (lay persons). Between 8-12 people usually serve on an AWERB. |
| National ethics committee | [Animals in Science Committee](https://www.gov.uk/government/organisations/animals-in-science-committee) (ASC) provides independent advice to the Home Office Inspectors and Secretary of State. The committee consists of up to 12 members with relevant expertise in animal welfare and care, scientists, those with veterinarian training and lay persons. |
| Other committees provide advice? | A subgroup of Home Office Inspectors (HOIs) with NHP expertise linked to Animals in Science Regulation Unit (ASRU) provide advice and can suggest changes or request further justifications for the use of procedures. |
| What do they consider during their approval process? | |
| 3Rs implementation | ‘Under the Animals (Scientific Procedures) Act 1986 section 5B(3)(b), in carrying out the evaluation of the programme of work, the Secretary of State must assess the compliance of the programme of work with the principles of replacement, reduction and refinement. These principles are described in section 2A(2) of the Act.’ Copy from UK Home Office Project Licence application form.  In addition, the evaluation includes justification for the species and the numbers of animals used, including appropriate power analysis. Explain refinements to procedures used and actions to be taken, and justify, if necessary, why available refinements may not be used. Discussion on whether reduction and/ or replacement techniques are available or not. |
| Project authorisation | Regular meetings with the NVS and NACWO are held to ensure project is on course and if any changes have to be implemented. May need protocol and procedure changes to project licence. These changes require approval from AWERB, and sometimes ASC. They also require authorization from HOI and sign off from Home Office Secretary of State.  AWERB is also responsible for the retrospective analysis half way through the project (typically at the two-year stage) and at the end of the project. These retrospective reviews ([guide here](https://www.health-ni.gov.uk/publications/animal-scientific-procedures-retrospective-assessment-guidance)) are then submitted to the Home Office.  The regional Home Office Inspector will undertake regular, unannounced visits and may access any records, if requested. |
| Harm/Benefit analysis | The costs to the animal in relation to the perceived benefits are used as a basis for deciding if the project licence can be approved. This analysis is conducted by the AWERB and at the national level by ASRU and the HOIs. [ASRU guidance](https://assets.publishing.service.gov.uk/government/uploads/system/uploads/attachment_data/file/487914/Harm_Benefit_Analysis__2_.pdf) is available here. Harm/benefit assessments are also embedded in the retrospective analyses of the project. |

**Table 1B. Overview of ethical regulations for conducting neuroscience procedures in non-human primates (macaques and marmosets) in France.**

| **France** | |
| --- | --- |
| What committees review and approve an NHP application? | |
| Local ethics committee | The local ethical committees (CEEA) provide an ethical evaluation of the NHP project applications and a recommendation. If the local ethical committee does not approve the NHP project application, the application is not forwarded to the National ethics evaluation level.  They have to comply to the National policy on animal research, issued by the National steering committee on animal research (CNREEA) and abide to the national decrees relating the ethical evaluation and the authorization of projects involving the use of animals in experimental procedures and their updates.  They have to be registered to the secretariat of the National steering committee on animal research (CNREEA), hosted by the Ministry of higher education, research and innovation.  They commit to operate in a transparent and independent manner, to render their judgment with complete impartiality and to guarantee the confidentiality of the files submitted to them.  Local ethical committees are at minimum composed of a veterinary, a scientist involved in animal research (NHP research when relevant), a technician involved in support to animal research (NHP research when relevant), an animal caregiver and a person naïve to animal research (lay person). Eight to 10 people usually serve on local ethical committees. |
| National ethics committee | Group of experts, nominated by the Ministry of higher education, research and innovation, in charge of evaluating elementary compliance and issuing the final approval, on the basis of the initial screening by the local ethical committees.  This group of experts works in close interaction with the National steering committee on animal research (CNREEA). This group of experts is composed of five experts nominated by the Ministry of higher education, research and innovation and the Ministry of Agriculture, and is composed of scientists and technicians with expertise in animal research. |
| Other committees provide advice? | Yes. The National steering committee on animal research (CNREEA) issues recommendations and enforces the ethics and deontology of animal research, and promotes all methods that are susceptible of improving animal welfare in animal research.  The National steering committee on animal research (CNREEA) is composed of scientific experts in animal research from the public sector, scientific experts in animal research from the private sector, experts from the human medical field, veterinaries, experts in philosophy, law, sociology and animal welfare representatives. |
| What do they consider during their approval process? | |
| 3Rs implementation | Yes. This involves justifying the number of animals used, including an appropriate power analysis. Explanations are required for refinement actions taken and justify if necessary why available refinement actions may not be taken. There is also consideration about whether replacement techniques are available or not. |
| Project authorisation | Yes. An animal welfare body is constituted in every research center, composed of research center appointed veterinary, scientists and technicians.  It is responsible for the continuous check on project progress relative to project authorization, approving minor changes in the research project and referring to the local ethical committee in case of major change.  It is responsible for the retrospective analysis at the end of the project, together with the local ethical committee (CEEA).  Meetings and efficiency of Animal welfare bodies is controlled once a year by departmental state inspection.  Annual departmental state inspection (DDPP) verifies that all ongoing research projects are properly authorized, implemented and retrospectively evaluated. |
| Harm/Benefit analysis | There is no explicit harm benefit assessment. However, harm/benefit assessment is at the root of ethics. It is thus embedded in the 3R assessment as well as in the scientific justification of the project.  Harm/benefit assessment is also embedded in the retrospective analysis performed at the end of the project. |

**Table 1C. Overview of ethical regulations for conducting neuroscience procedures in non-human primates (macaques and marmosets) in China.**

| **China** | |
| --- | --- |
| What committees review and approve an NHP application? | 2 levels of review: Primate IACUC committee in the institute and University IACUC committee at medical school |
| Local ethics committee | Primate IACUC committee: Head vet + IO + 4 PIs + 1 person from the community who is not a scientist or involved in research. 1 secretary to record.  University IACUC committee: 24 members including vice president, director of facilities, director of animal facilities, head veterinarian, and PIs from medical school, other colleges, and hospitals. |
| National ethics committee | No |
| Other committees provide advice? | Yes. University IACUC. MRI Center Protocol Committee. |
| What do they consider during their approval process? | |
| 3Rs implementation | Yes. Regular training courses are offered that every user must take. This includes 3R’s (Animal Welfare Act, Guide to Lab Animals), animal husbandry, animal use (surgery, anesthesia, EEG, behavioral training, water regulation, animal endpoint criteria), biosafety, personnel safety and PPE, and protocols and forms, and compliance. 3R’s: Justification for use of NHPs, for number of NHPs, and procedures to minimize distress are addressed in each PIs protocol. |
| Project authorisation | Yes. Protocols and amendments are thoroughly reviewed; responses to any questions must be addressed in writing by PI and re-reviewed by committee. Compliance consists of unannounced inspections of labs (once per year). Failure to address non-compliance is met with removal of privileges (animal facility keycard inactivated, no approval to purchase animals or drugs). If there is a sustained pattern of non-compliance, the user will be barred from the institute. |
| Harm/Benefit analysis | The procedures we conduct which can be considered ‘harmful’ are surgery and anesthesia, single housing, and water regulation. Surgical, anesthesia, and analgesia procedures are well established. Justifications for single housing (Evaluate social benefit, do animals fight when housed together? What is the risk for animal health? What is the need for project goals?). Water regulation (standard is 15% reduction of body weight is met with increased scrutiny and monitoring; 20% reduction in body weight the animal is taken off study). |

**Table 1D. Overview of ethical regulations for conducting neuroscience procedures in non-human primates (macaques and marmosets) in the US.**

| **USA** | |
| --- | --- |
| What committees review and approve an NHP application? | |
| Local ethics committee | Yes. Local IACUCs are required by PHS regulations and the Animal Welfare Act to consist of at least 5 members.  One veterinarian, one practicing scientist experienced in animal research, one member whose primary concerns are non-scientific and one non-affiliated member  <https://olaw.nih.gov/resources/tutorial/iacuc.htm> |
| National ethics committee | No |
| Other committees provide advice?^3^ | No. Given the considerable expenses involved NHP research is typically funded by a federal agency. As part of the application, a section on vertebrate animals has to be filed, which contains all relevant information on the procedures that the laboratory animals will undergo. The review panel discusses this section along with the application. If the procedures detailed in the application do not meet ethical standards, it will not get funded. Approval by the local IACUC is necessary in order to receive federal support. |
| What do they consider during their approval process? | |
| 3Rs implementation | Local animal research protocols usually contain a question asking about how the PIs’ selection of the model considers the 3Rs and often require a detailed literature research documenting the necessity of the animal model. |
| Project authorisation | By Federal regulation, IACUCs are charged with biannual inspections of animal housing and animal use areas. Post approval monitoring of procedures happens in this context. This is an ongoing issue at some universities – how to do post IACUC protocol approval monitoring of procedures. USDA covered species (all warm-blooded animals except rats, mice and birds) protocols are reviewed every year. All others are reviewed every 3 years. |
| Harm/Benefit analysis | The IACUC members discuss the harm and benefits of the research at the time of protocol review. This is always done for protocols involving pain that is difficult to relieved or for animals expected to become sick or otherwise impaired as a result of the scientific procedure. For some IACUCs, this is also assessed for any experimental procedure. |
| Anything else, not covered above? | Animal research in the US is governed by Federal legislation – the Animal Welfare act and the enforcement branch of the USDA called APHIS oversees animal use and research. Biomedical research funded by the NIH and NSF or DOD is under additional regulations as outlined by the PHS of NIH and the enforcement branch OLAW. FDA also provides oversight for its supported research.  AAALAC International is a private, non-profit organization that promotes the humane treatment of animals used in scientific research. AAALAC standards exceed those of federal laws and policies, and accreditation by AAALAC International is a clear demonstration of an institution's commitment to the responsible treatment of animals. Many US institutions are AAALAC accredited. |

**Table 2A. Welfare and other regulations regarding accommodation, care and use of NHPs (macaques and marmosets) in the UK.**

| **UK** | |
| --- | --- |
| Use of wild NHPs for research purpose | Banned |
| Inspections (min. number) | Once a year |
| Single housing | Only in exceptional circumstances. Must be approved by vet and animal welfare officer. Regularly assessed for stereotypies and overall well-being and extra enrichment must be provided. Must still have visual, auditory, and smell of other monkeys. |
| Limits on pain, suffering and distress | Must be defined in protocols and for each procedure. Needs approval and helps to formulate the harm/ benefit analysis |
| Killing method | Anesthetic overdose |
| Weaning age macaques | At least 8 months |
| Weaning age marmosets | At least 8 months |
| Cage size for macaques in experiments | |
| - - Min. volume per adult animal (> 3yrs) | 1.8 m^3^ |
| - - Min. enclosure height | 1.8 m |
| Cage size for marmosets | |
| - - Min. volume per adult animal (> 5months) | 0.2 m^3^ |
| - - Min. enclosure height | 1.5 m |
| Double-tiered cage | Allowed as ‘outpens’ but NHPs can’t live in double-tiered caging only for long periods of time |
| Grid floor | Not allowed in long term. Exceptions for a short-time allowed on compelling scientific or veterinary reasons (e.g. first 24 hours after neurosurgery). |
| Physical enrichment | Should be present |
| Fluid control | Allowed and to follow guidelines in Prescott et al. (2010). Use either minimum volume: 20mls /kg/ day or  minimum time: at least 3-6 hours per 24 hours. Require one day of *ad libitum* per week. Regular weekly weights needed. |
| Food control | Allowed and to follow guidelines in Prescott et al. (2010)  If food is given at specific times (i.e. after experimental testing/ training) then minimum weighed amount of high calorific protein mash is required based on weight and age of monkey. Must weigh the monkeys at least every two weeks. Always have to be gaining/ stabilized weight otherwise intervene if weight drops 10% from original. |
| Capture | Restricted with use of pole and collar (i.e. it is not allowed as the only option in the UK). Use of positive reinforcement training techniques (PRT) are necessary. |
| Restraint | Time limits on head-posting: up to 5 hours (neurophysiology); up to 2-3 hours (awake MRI).  Typically, on procedures 4-5 days per week; not allowed 6 days of max restraint in a row. Can’t work 7 days in a row. |
| Ongoing training | Annual primate welfare meeting and expectation is to attend on a regular basis. |

**Table 2B. Welfare and other regulations regarding accommodation, care and use of NHPs (macaques and marmosets) in France.**

| **France** | |
| --- | --- |
| Use of wild NHPs for research purpose | Banned |
| Inspections (min. number) | Once a year |
| Single housing | Only in exceptional circumstances. Must be approved by vet and animal welfare body.  Must maintain social contact (i.e. visualizing, hearing, smelling other monkeys).  Regularly assessed for stereotypies and overall well-being and extra enrichment must be provided. |
| Limit of pain, suffering, and distress | Must be defined in protocols and for each procedure.  This is an important item of project authorization.  Requires discussion and decision by animal welfare body. |
| Killing method | Anesthetic overdose |
| Weaning age macaques | At least 8 months |
| Weaning age marmosets | At least 6 months |
| Cage size for macaques on experiments | |
| - - Min. volume per adult animal (> 3yrs) | 1.8 m^3^ |
| - - Min. enclosure height | 1.8 m |
| Cage size for marmosets | |
| - - Min. volume per adult animal (> 5months) | 0.2 m^3^ |
| - - Min. enclosure height | 1.5 m |
| Double-tiered cage | NHPs can’t live in double-tiered caging  Allowed by animal welfare body for short periods due to health or experimental requirements |
| Grid floor | Not allowed |
| Physical enrichment | Should be extensive, include novelty and regularly assessed by animal welfare body |
| Fluid control | Allowed as long as animal welfare is assured as assessed by animal welfare body.  Must weight monkey daily. Working weight should be stable.  In any case, EU directive 2010/63, i.e. no less than 20ml/kg/day and one day off is a requirement.  Best practice enforced by animal welfare body should follow the CRNEEA recommendation on the matter, based on the GDR Biosimia recommendation (a research group on NHPs in biomedical research), NC3Rs recommendations, Prescott et al., 2010, Gray et al., 2016, Gray et al., 2019. |
| Food control | Allowed as long as animal welfare is assured as assessed by animal welfare body.  Must weigh monkey at least twice a week. Working weight should be stable.  Post-experimental training food complement should be high protein food, and should be weighed to achieve the trade-off between motivation and minimal daily caloric intake. |
| Capture | Squeeze-back cages, nets, and pole-and-collar, based on positive reinforcement and clicker-training. |
| Restraint | Time limits on head-posting: up to 6 hours (neurophysiology); up to 2-3 hours (awake MRI)  Typically, 4-5 days. Can’t work more than 5 days in a row. |
| Ongoing training | The GDR Biosimia, a research group on NHPs in biomedical research focusses on the 3R and animal welfare. Expectation is to attend annual meetings on a regular basis.  All participants in NHP research or care have to comply to the EU Directive 2010/63 (minimum of 3 days training in 6 years). Animal welfare bodies reinforce regular attendance to training events in France and Europe. |
| Other? Please provide details. | Pre-determined humane endpoints are defined in the authorized project. This also applies to euthanasia.  Animal welfare bodies discuss the issue and have with the veterinarian the final say.  NHPs can be reused if retrospective evaluation of cumulative experimental severity is favourable in accordance with the EU Directive. Animal welfare bodies discuss the issue and have, with the veterinarian, the final say.  All French governmental biomedical institutions using NHP (CNRS, INSERM, CEA) are members of AALAC. |

**Table 2C. Welfare and other regulations regarding accommodation, care and use of NHPs (macaques and marmosets) in China.**

| **China** | |
| --- | --- |
| Use of wild NHPs for research purpose | Banned, now strongly enforced since coronavirus pandemic |
| Inspections (min. number) | Once a year |
| Single housing | Pair housing in roughly half of macaque monkey population. Others are approved exemptions by IACUC committee. Must maintain social contact (i.e. visualizing, hearing, smelling other monkeys). Vet assesses for stereotypies and well-being. Enrichment (variety of foods, toys, music, tv, behavioral training) is provided. Marmosets live in cages that house families. |
| Limit of pain, suffering, and distress | All surgical procedures are conducted under surgical plane of anesthesia. Stereotypies or other behavioral indications of distress are assessed for treated either medically, with enrichment, or with social pairing methods. |
| Killing method | Anesthetic overdose |
| Weaning age macaques |  |
| Weaning age marmosets |  |
| Cage size for macaques in experiments | |
| - - Min. volume per adult animal (> 3yrs) | 1.0 m x 0.9 m x 0.7m |
| - - Min. enclosure height | 0.8m |
| Cage size for marmosets | |
| - - Min. volume per adult animal (> 5months) | No national standard, our facility uses 1.0m x 1.0m x 2.5m cages for a family |
| - - Min. enclosure height | 2.5m |
| Double-tiered cage | Animals rotate through large play cages |
| Grid floor |  |
| Physical enrichment | Yes |
| Fluid control | Allowed. Must justify and explain methods of monitoring health and welfare. Weigh daily. 15% weight loss from baseline, must be reviewed by vet. 20% weight loss from baseline, take off study. One day off per week. 20ml/kg is our guideline. |
| Food control | Yes. |
| Capture | Either pole and collar or, for trained monkeys, jump into chair  Initial training involves encouragement with food, water, juice, monkey learns to trust trainer |
| Restraint | Head-posting up to 4 hours/day for neurophysiology, up to 2 hours per day for MRI |
| Ongoing training | Once per year |
| Other? Please provide details. | Animals take turns in large play cage, frequency depends on animal usage, but typically once every 2 weeks |

**Table 2D. Welfare and other regulations regarding accommodation, care and use of NHPs (macaques and marmosets) in the US.**

| **USA** | |
| --- | --- |
| Use of wild NHPs for research purpose | Not banned – especially not field biology work  Purchase for biomedical research is strongly encouraged from reputable sources. |
| Inspections (min. number) |  |
| Single housing | Scientific justification exceptions allowed  Or veterinary exceptions allowed (e.g. no suitable pairs). |
| Limits of pain, suffering, and distress | Required unless scientifically justified and requires approval by IACUC. |
| Killing method | Follows AVMA guidelines |
| Weaning age macaques |  |
| Weaning age marmosets |  |
| Cage size for macaques in experiments | Depends on weight of the animal. For a monkey up to 15 kgs |
| - - Min. volume per adult animal | Floor size at least 6 feet square (0.56 m^2^) |
| - - Min. enclosure height | At least 32 inches (0.81m) high |
| Cage size for marmosets |  |
| - - Min. volume per adult animal (> 5months) |  |
| - - Min. enclosure height |  |
| Double-tiered cage |  |
| Grid floor |  |
| Physical enrichment | Enrichment plans are required per institution. These include psychological enrichment as well as exercise / physical enrichment. |
| Fluid control | Allowed with scientific justification and approval by IACUC.  USDA guidelines require at least two times a day access to fluid. This requirement is often exempted on a protocol by protocol basis with scientific justification. Typically, use 20- 22ml/kg minimum. Documentation and careful weighing and monitoring of the animals is required. Use of animals preferred fluids in lieu of restriction is encouraged. Minimum amount of restriction necessary to perform tasks also encouraged. |
| Food control | Same as fluid. |
| Capture | As needed. Use of pole and collar with appropriate habituation and training. |
| Restraint | Scientific justification required and approval of IACUC.  Up to 12 hours (otherwise considered housing) |
| Ongoing training | Individual PI initiated unless there are issues and the veterinarian together with the IACUC requires. |

References

Gray, H., Bertrand, H., Mindus, C., Flecknell, P., Rowe, C., Thiele, A., 2016. Physiological, Behavioral, and Scientific Impact of Different Fluid Control Protocols in the Rhesus Macaque (Macaca mulatta). *eNeuro*, 3, ENEURO.0195-16.2016.

[doi.org/10.1523/ENEURO.0195-16.2016](https://doi.org/10.1523/ENEURO.0195-16.2016)

Gray, H., Thiele, A., Rowe, C., 2019. **Using preferred fluids and different reward schedules to motivate rhesus macaques (Macaca mulatta) in cognitive tasks**. *Lab Anim*, 53, 372-382, doi:[10.1177/0023677218801390](https://doi.org/10.1177/0023677218801390)

Prescott, M.J., Brown, V.J., Flecknell, P.A., Gaffan, D., Garrod, K., Lemon, R.N., Parker, A.J., Ryder, K., Schultz, W., Scott, L., Watson, J., Whitfield, L., 2010. Refinement of the use of food and fluid control as motivational tools for macaques used in behavioural neuroscience research: report of a Working Group of the NC3Rs. *J Neurosci Methods*. 193, 167-188. doi:10.1016/j.jneumeth.2010.09.003
